# Microstructural Evolution of a 3003 Based Aluminium Alloy during the CSET Process

**DOI:** 10.3390/ma14195770

**Published:** 2021-10-02

**Authors:** Orsolya Molnárová, Stanislav Habr, Esther de Prado, Jaroslav Čapek, Ondřej Ekrt, Gergely Németh, Přemysl Málek, Pavel Lejček

**Affiliations:** 1 Institute of Physics, Czech Academy of Sciences, Na Slovance 2, 18221 Prague, Czech Republic; habr@fzu.cz (S.H.); prado@fzu.cz (E.d.P.); capekj@fzu.cz (J.Č.); ekrt@fzu.cz (O.E.); lejcekp@fzu.cz (P.L.); 2 Department of Physics of Materials, Charles University, Ke Karlovu 5, 12116 Prague, Czech Republic; nemeth@ujf.cas.cz (G.N.); malek@met.mff.cuni.cz (P.M.); 3 Department of Neutron Physics, Nuclear Physics Institute of the CAS, Husinec-Řež 130, 25068 Řež, Czech Republic

**Keywords:** tube forming, complex shearing of extruded tubes, severe plastic deformation, microstructure, microhardness

## Abstract

A new severe plastic deformation technique, known as the complex shearing of extruded tube (CSET), was applied to a 3003 based model aluminium alloy. This technique, consisting of a combination of extrusion and two consecutive Equal Chanel Angular Pressing (ECAP) passes accompanied with concurrent torsional straining, is capable to produce a fine-grained tubular sample directly from a bulk metallic cylinder in one forming operation. In the present paper, the microstructural development of the alloy during partial processes of CSET was studied in detail using light microscopy, electron backscatter diffraction, and transmission electron microscopy. It was found that CSET technique refines the grain size down to 0.4 µm and, consequently, increases the microhardness from the initial value of 40 HV to the final value of 120 HV. The contributions of partial processes of CSET to the total strain were estimated.

## 1. Introduction

Aluminium alloys belong to the materials widely used in engineering applications. Due to the increasing requirements on the material properties in most devices, there is a continual demand for strength elevation of aluminium alloys. Remarkable strengthening can be achieved by a proper heat treatment or by application of severe plastic deformation (SPD) methods [1]. Equal channel angular pressing (ECAP) is a widely known SPD method, where the intense plastic straining through a simple shear is capable to strengthen the materials and results in a massive microstructural refinement [2]. Similarly, high pressure torsion (HPT) subjects the samples to high torque under high pressure leading to submicrometre-sized grains along with enhanced strength [3]. These and various other SPD methods were developed to produce high-strength bars or sheets. However, there is a wide application field also for the tubular samples, which cannot be readily processed by ECAP or HPT. Therefore, these SPD methods have to be adapted for that purpose.

Several researchers processed tubular samples by traditional ECAP using sand as mandrel during procession, which filled the tube and ensured its shape preservation. They reported about enhanced tensile strength and hardness in the samples [4,5]. Another variation is named as tube channel pressing (TCP) during which the tube is pressed through a tubular channel with a neck zone [6]. During parallel tubular channel angular pressing (PTCAP), the already tubular sample is held between an inner and outer die and the tubular punch presses the tube through a channel with two shear zones [7], whereas tubular channel angular pressing (TCAP) [8], which works on a similar basis as PTCAP, utilizes alternating pressing through a tubular channel with three shear zones.

Other SPD techniques were created by modification of HPT to exploit the high hydrostatic pressure during straining. The methods named tube high-pressure shearing (t-HPS) [9,10] or high pressure tube twisting (HPTT) [11,12] both apply large hydrostatic pressure on the tubular sample placed between an inner and outer mandrel. The external torque through the rotation of one or both mandrels then lead to shear deformation resulting in strengthening through grain refinement in the tube wall. However, as these methods deform the whole volume of the body, there is a serious length limitation of samples. This length restriction was overcome in high pressure tube shearing (HPTS) method, where the main deformation zone was limited [13]. During HPTS, the tubular sample is drawn through a rotating die, whereas the wall thickness of the tube is reduced by an internal rotating mandrel. The shear deformation due to the rotation and high local hydrostatic pressure lead to fine grain size and gradient microstructure [13]. These methods generally combine several press steps for an incremental straining to achieve the required strength level.

All above mentioned methods start with already tubular samples. A recently introduced processing method called complex straining of extruded tube (CSET) prepares ultra-fine-grained tubular sample with enhanced strength from a cylindrical bulk sample in one step [14]. The combination of tube extrusion followed by two consecutive ECAP passes and concurrent torsional straining from the rotation of the mandrel was demonstrated to result in remarkable hardness enhancement through grain refinement [14].

The goal of the present paper is to discuss the individual deformation processes occurring in particular stages of CSET, to estimate the resulting strain, and to compare it with other methods. To investigate the effect of individual processing stages, CSET was applied to produce a tubular sample from a 3003 based model aluminium alloy. The microstructural evolution caused by each stage of deformation was analysed and the gradual strengthening was studied by microhardness measurement.

## 2. Materials and Methods

A 3003 based model aluminium alloy used in this work was prepared by the melting of high-purity Al, Mn, and Cu in a vacuum induction furnace VSG-02 (Balzers AG, Balzers, Liechtenstein) in a graphite crucible under argon atmosphere. The exact chemical composition of the used alloy was tested with an Ametek EDAX Orbis X-ray Fluorescence analyser (Ametek EDAX, Weiterstadt, Germany) which revealed a composition of 0.12 wt.% of Cu, 1.2 wt.% of Mn, and Al as balance. The alloy was soft annealed at 450 °C for 4 h and furnace cooled. The phase composition of the sample was determined by X-ray diffraction (XRD) techniques. The measurements were carried out on an X’Pert Pro PANalytical powder diffractometer (Malvern Pananalytical Ltd., Royston, UK) using Co Kα X-ray radiation with an Fe Kβ filter. The patterns were collected using Bragg–Brentano geometry and subsequently processed by X’Pert HighScore Plus software with access to the PDF4 database in order to perform phase identification, and by Topas V3 software in order to make Rietveld refinement.

**Figure 1:**
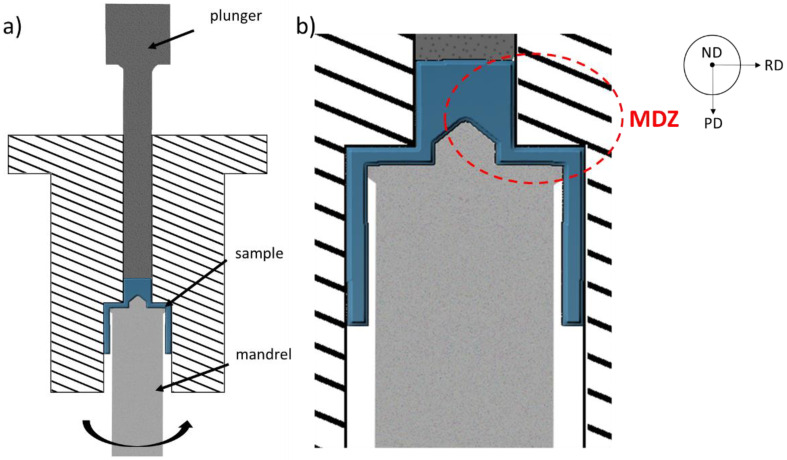
Scheme of the CSET device (**a**) and its detail (**b**) showing the main deformation zone (MDZ). PD—pressing direction, RD—radial direction, ND—normal direction.

The cast ingot was machined into cylindrical bars with the length of 55 mm and the diameter of 11 mm. The bars were processed by CSET. The scheme of the device is shown in Figure 1. The lubricated bar was pressed through the device at room temperature (RT) by a plunger moving with the translational velocity of 0.2 mm s^−1^ under simultaneous rotation of the mandrel with the frequency of 0.2 Hz (12 rpm). The sample was not fully pressed through the die in order to study the evolution of the microstructure in individual stages of deformation. Therefore, the CSET process was stopped in the state schematically presented in Figure 1. The resulting tube had the outer diameter of 26 mm and the wall thickness of 2 mm. The production details are described in detail in [14].

The grain size of the annealed original bar and the flow lines of the CSET processed samples were studied by light microscopy (LM; Zeiss Axio Observer D1m microscope (Carl Zeiss Microscopy GmbH, Jena, Germany)). To reveal these microstructural features, the samples were fully polished and etched by a modified Dix–Keller reagent. Scanning electron microscopy (SEM; FEI Quanta 3D FEG) (FEI Czech Republic s.r.o., Brno, Czech Republic) and transmission electron microscopy, along with scanning transmission electron microscopy (TEM, STEM; FEI Tecnai TF20 X-twin) (FEI Czech Republic s.r.o., Brno, Czech Republic), were utilized to reveal microstructural details. Electron backscatter diffraction (EBSD) measurements were performed in SEM on samples, which were electrolytically polished using a 10% HClO_4_ in ethanol solution (10 V, 120 s). Samples for TEM were prepared by ion milling using PIPS (Gatan, 5 keV, 5° and 3°) or using focused Ga ion beam (FIB) in SEM for preparation of samples from specific small areas.

The Vickers microhardness measurement was used to investigate the mechanical properties of CSET-treated materials using the Q10A+ microhardness tester (ATM Qness GmbH, Golling, Austria) under a load of 0.49 N (HV0.05). The distance between individual indents was 200 µm, and each indent was held for 10 s.

## 3. Results

### 3.1. Microstructural Evolution of the 3003 Alloy during Individual Stages of the CSET Process

As is apparent from Figure 1, at the beginning of the CSET process, the full bar is extruded into a tube with the wall thickness of 2 mm. In the next step, the tube is pressed through two consecutive rectangular ECAP channels. Concurrently, additional deformation can be initiated by the rotational movement of the mandrel. All these deformation steps occur in a particular limited area denoted as the main deformation zone (MDZ) in Figure 1b. Below this area, the diameter of the rotating mandrel is decreased and the sample is no more affected by it.

**Figure 2:**
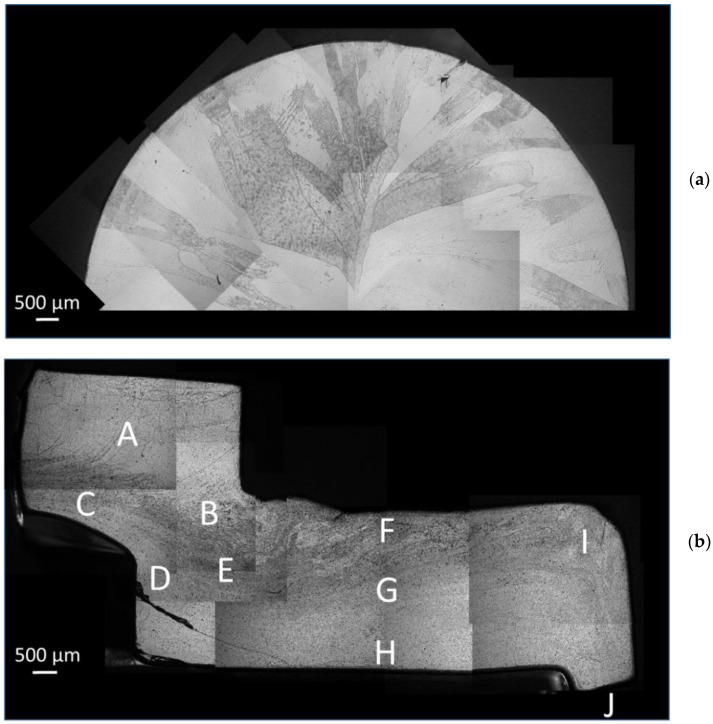
LM figure of the initial state showing (**a**) large elongated grains and (**b**) flow lines visible on the etched surface of the cross-section on the ND plane in the MDZ area after CSET. For the meaning of areas A–J, see the text and Table 1.

The microstructure of the annealed starting sample is presented in Figure 2a. Chemical etching revealed the initial grain structure with coarse grains several millimetres long and a few hundred micrometres wide. The same chemical etchant was used to reveal the flow lines in the CSET processed sample. Figure 2b shows the flow lines in the MDZ on the cross-section cut parallel to the tube axis on plane ND (normal direction—see Figure 1b). A gradual refinement of the microstructure in the MDZ is readily visible. In accordance with the apparent contrast variation in the MDZ, several positions in the MDZ (Table 1) were chosen to study the microstructural evolution during particular deformation steps.

At position A, the material is before extrusion and affected only by a pressure applied with the plunger. The microstructure consists of grains with a size up to several hundreds of micrometres, similar to that in the annealed sample (Figure 2a). The microstructure at position B shows a heterogeneous contrast reflecting a high variation in grain boundary density. The contrast of the LM figure at positions C and D close to the rotating mandrel reveals a refined microstructure after extrusion. Position E documents the microstructure of the material going through the first ECAP pass. The area between two ECAP passes exhibits different contrasts through the wall. Whereas a relatively high variation of contrast at the outer side (positions F and I) suggests a heterogeneous microstructure, a rather reduced contrast at the inner side close to the rotating mandrel (position H) reveals a homogeneous refined structure. A similar reduced contrast was observed in the middle of the tube after the second ECAP pass (position J).

The black crack visible at position D was most probably caused during the last seconds of the sample processing or during the dissemble of the CSET device aimed to remove the sample, as the crack was shallow and localized only to this part of the whole tubular sample. No other cracks were found in the CSET-processed tubular sample.

**Figure 3:**
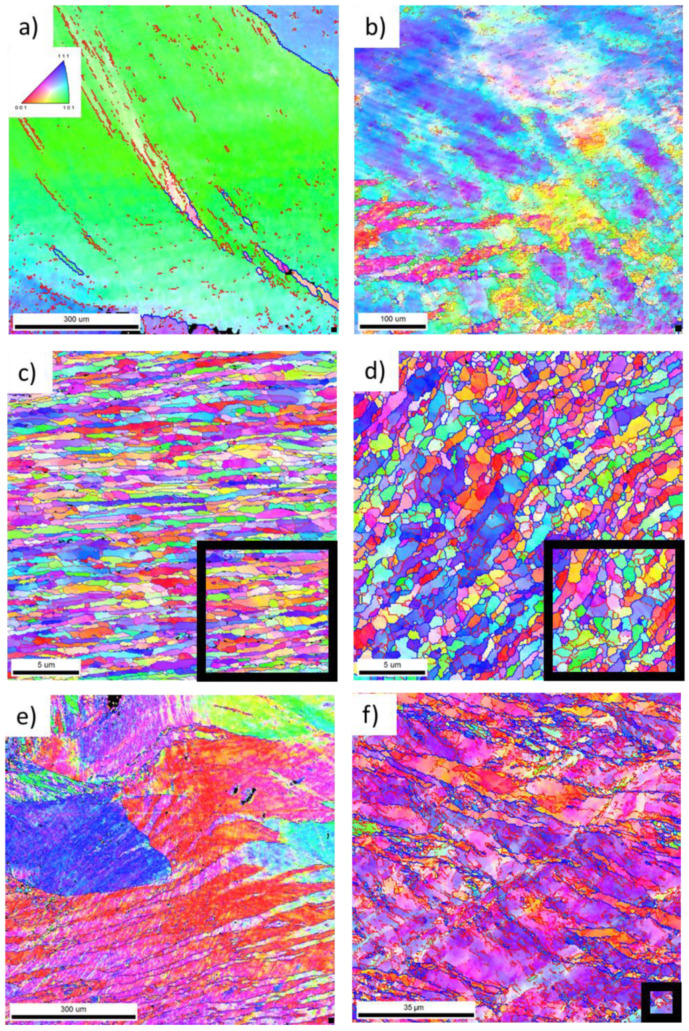

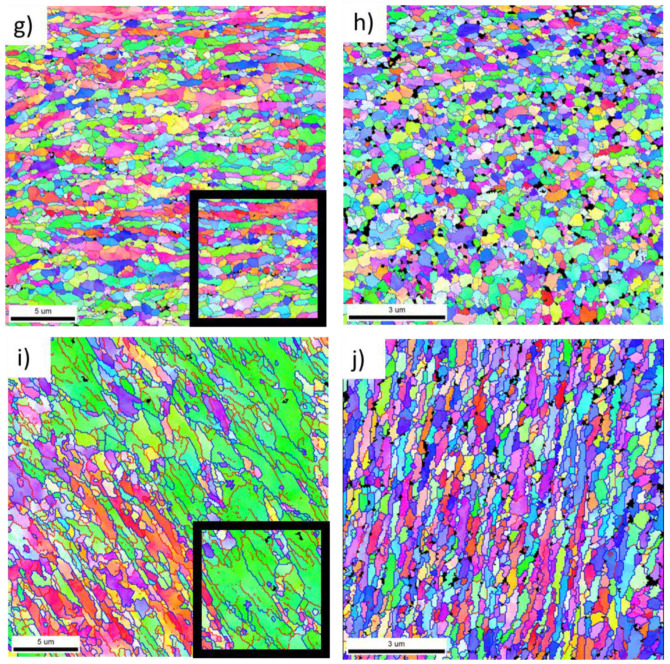
Orientation image maps (OIMs) reflecting the evolution of the microstructure during individual stages of CSET process. The studied areas and their designation (**a**–**j**) correspond to those marked in Figure 2b (A–J, respectively) and in Table 2. The orientation of the OIMs relative to the setup is the same as presented in Figure 1 and Figure 2. Black squares in the right bottom corner in OIMs (**a**–**g**,**i**) represent the size of the smallest maps in (**h**,**j**).

To investigate the course of the CSET process and to quantify the degree of the microstructural refinement occurring during its particular stages, EBSD measurements were performed at all positions denoted in Figure 2b. The corresponding orientation image maps (OIMs) are presented in Figure 3. High-angle grain boundaries (HAGBs, misorientation above 15°) and low-angle grain boundaries (LAGBs, misorientation between 5 and 15°) are highlighted by blue and red colours, respectively. In order to reflect the true microstructure at each studied position, the map size and step size of OIM had to be modified in each area. The step size was reduced so that several measurement points would fit in one grain, and the map size was then reduced to limit the time of acquisition to a reasonable value. In order to simplify the comparison of the OIMs from the different areas, black squares in the right bottom corner in OIMs (Figure 3a–g,i) represent the size of the smallest maps in Figure 3h,j. The orientation of the maps relative to the experimental set up is the same as presented in Figure 1 and Figure 2. At some positions, the microstructure is extremely inhomogeneous and any evaluation of the mean grain size and distribution of misorientation angles is not possible. At positions where the microstructure was relatively homogeneous, the mean grain size *d* was evaluated from the mean intercept *l* as *d* = 1.74 *l* [15]. This method is very useful, especially in cases when the grains are elongated along a specific direction as it enables us to determine also the grain aspect ratio. Simultaneously, the proportion of HAGBs was determined. All these characteristics are listed in Table 2.

Figure 3a taken from the area at position A before extrusion shows large grains with only several LAGBs inside, i.e., a preservation of initial microstructure. Figure 3b documenting the area at position B after extrusion and far from the rotating mandrel reveals no grain refinement; however, the grain interiors are more deformed, as reflected by increased colour variations inside the grains. Much finer and elongated grains were observed after extrusion at positions C and D in the vicinity of the rotating mandrel (Figure 3c,d). This means that the shear deformation caused by the mandrel rotation accelerates the process of grain refinement. The direction of grain elongation coincides well with the direction of material flow. The width of the grains was close to 0.8 μm and their length exceeded 4 μm so that a large aspect ratio close to 5 was observed at position C. Numerous elongated grains were divided by LAGBs into subgrains. The analysis of the misorientation angles showed that nearly 80% of boundaries in this area were of high angles.

During the first ECAP pass, a large strain is introduced into the material. The OIM taken from position E in the middle of the tube wall reveals highly deformed grains reflected by high colour variations inside the grains in Figure 3e. A large majority of grain boundaries are of low-angle character; less than 20% of the boundaries have misorientations larger than 15°. This corresponds to the initial stages of severe plastic deformation. The evaluation of the grain size is not possible at this stage.

Positions F, G, and H correspond to the material after the first ECAP pass. The corresponding OIMs reveal a gradient microstructure at this stage of CSET. The OIM at position F, far from the mandrel (Figure 3f), is similar to that at the position E, i.e., the original large grains are strongly deformed and the majority of the grain boundaries in this area are LAGBs. The proportion of HAGBs reaches the value of 35%. In the middle of the wall, a refined microstructure is found (Figure 3g). Most grains are elongated along the horizontal direction and divided by LAGBs into equiaxed subgrains. The mean grain width is close to 1 µm and the mean grain length is close to 2 μm. The proportion of HAGBs exceeds 70%. The microstructure at position H, i.e., in vicinity of the rotating mandrel, is shown in Figure 3h. Further grain refinement to the value of 400 nm and a tendency to equiaxed grain shape is clearly visible in Figure 3h. The proportion of HAGBs reaches 90%.

The influence of the second ECAP on the microstructure at the outer side of the tube wall is visible from comparison of Figure 3f,i. The grains at position I are refined to several µm and exhibit a significant elongation in the shear direction. Simultaneously, the proportion of HAGBs increases from about 35% at position F to about 60% at position I. A remarkable grain refinement after the second ECAP pass was observed in the middle of the wall (position J). The grain size was reduced deeply below 1 µm and the proportion of HAGBs remained at 90%. The grains are clearly elongated along the direction of tube axis with the aspect ratio of 2.

Microstructural analysis performed at different locations of the MDZ thus revealed the gradual refinement of the grain structure due to individual deformation processes involved in CSET processing. With the ongoing deformation, the grain size gradually decreased, the grains became elongated, and the percentage of HAGBs increased to 90%. The shear deformation introduced through mandrel rotation accelerated the grain refinement.

**Figure 4:**
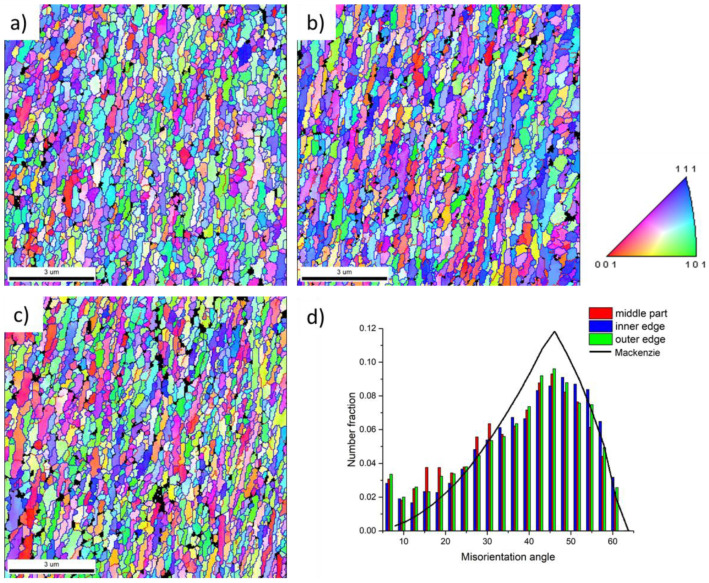
The microstructure of the final CSET-processed tube on the ND plane at different distances from the mandrel: (**a**) OIM at the inner tube surface, (**b**) in the middle of the wall, (**c**) at the outer tube surface, and (**d**) the distribution of misorientation angles (in degrees).

The microstructure of the final CSET processed tube was tested on the ND plane at different distances from the mandrel, i.e., at the inner tube surface (Figure 4a), in the middle of the tube wall (Figure 4b), and at the outer tube surface (Figure 4c). It can be seen that all microstructures are very similar, the mean grain width is close to 400 nm, and that the grains are elongated along the direction of the tube axis with the aspect ratio close to the value of 2. The proportion of HAGBs exceeds 90%. Figure 4d presents the distribution of misorientation angles, as evaluated from the OIMs in Figure 4a–c. These distributions are at all positions very similar and close to the random (Mackenzie) distribution denoted by the black line.

**Figure 5:**
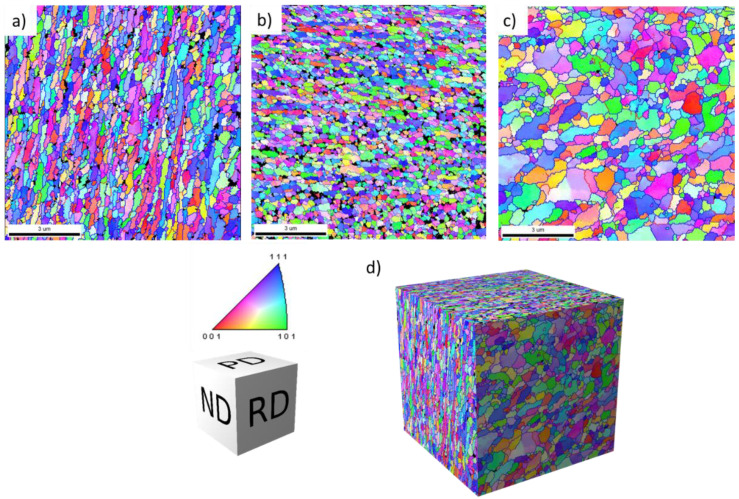
The microstructure of the completely CSET processed tube in the middle of the tube wall (position J in Figure 2) on three perpendicular planes. OIMs taken on plane ND (**a**), on plane PD (**b**), and on plane RD (**c**), along with the 3D reconstruction of microstructure (**d**). The IPF triangle represents the colours of the orientations in (**a**–**c**) and the schematic cube shows the orientation of the cube in (**d**).

The microstructure directionality in the final CSET processed tube was studied in the middle of the tube wall (position J in Figure 2). OIMs were taken from areas perpendicular to ND, RD, and PD directions. Figure 5a (as seen on ND plane) documents the elongation of grains along the direction of the tube axis (the OIM is identical with that in Figure 4b), whereas Figure 5b (as seen on PD plane) reveals elongation in the ND direction. Figure 5c (as seen on RD plane) shows a coarser grain structure with rather equiaxed grains on the RD plane. Figure 5d presents a 3D reconstruction of the microstructure to reflect the mutual orientation of the OIMs taken on three perpendicular planes. The data evaluated from OIMs thus reveal a pancake shape of grains with a smaller size close to 400 nm along the direction perpendicular to the tube axis. The proportion of HAGBs is close to 90% in all OIMs.

**Figure 6:**
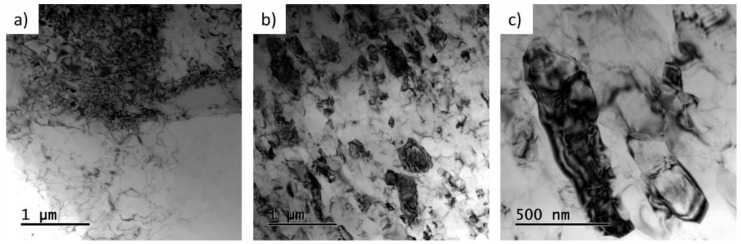
TEM micrographs presenting the microstructure of the sample at individual stages of CSET, (**a**) before extrusion, and (**b,c**) between the two ECAP passes (**b**,**c**), both RD.

Finer microstructural details were studied using TEM and STEM. The high density of dislocations was observed at position A before extrusion where the material was influenced only by the applied pressure (Figure 6a). The investigations performed in the area between the ECAP passes at position G revealed slightly elongated grains with a size up to 1 µm (Figure 6b). It can be seen at higher magnification that the grains are divided into subgrains (Figure 6c). The bend contour implies the presence of high level of internal stress in the grains.

**Figure 7:**
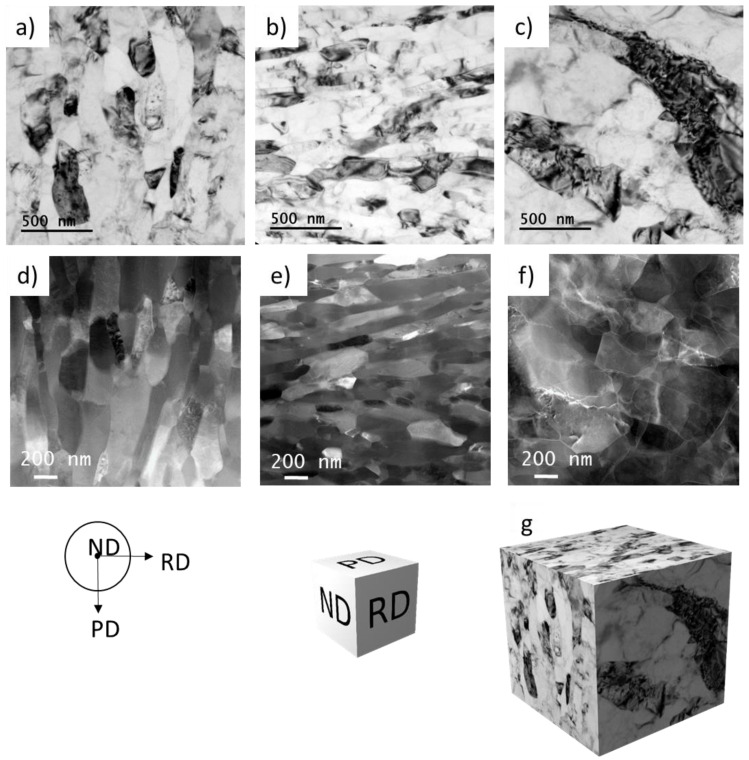
TEM (**a**–**c**) and STEM (**d**–**f**) micrographs presenting the microstructure in the middle of the tube wall in perpendicular directions, on ND (**a**,**d**), PD (**b**,**e**), and RD planes (**c**,**f**) of the tube wall, and 3D reconstruction of the grain structure (**g**).

Figure 7 presents TEM (a–c) and STEM (d–f) images of microstructure of the final CSET-processed tube in three perpendicular directions, analogous to the OIM maps in Figure 5. The cross-sections of ND (Figure 7a,d) and PD planes (Figure 7b,e) show elongated grains with a grain size less than 1 µm in the longer direction (Figure 7a,b). TEM analysis on RD plane revealed a coarser grain size and the grains were found to be rather equiaxed (Figure 7c,f). The 3D reconstruction of TEM microstructure from perpendicular directions on Figure 7g reflects the true shape of ellipsoidal grains. The majority of grains were visibly compressed in the radial direction.

**Figure 8:**
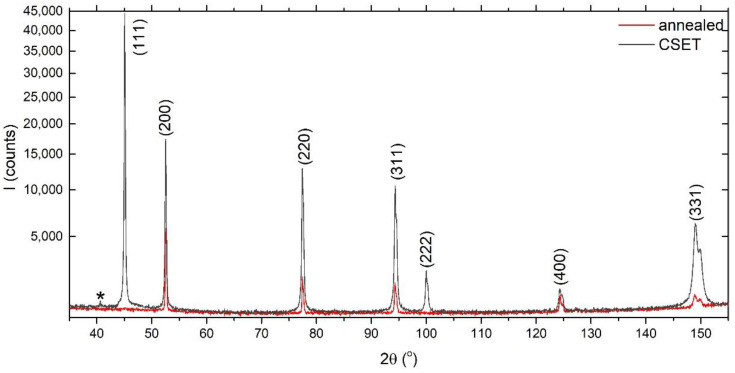
The X-ray diffraction pattern recorded from the annealed sample (in red) and from the tube wall on the RD plane of the CSET-processed sample (in black).

The microstructure development resulting from the CSET process was also studied by X-ray diffraction. Figure 8 shows the diffraction patterns recorded on the annealed sample and on the tube wall of the CSET-processed sample. Only the cubic (Fm-3m) phase was detected in both samples that correspond to Al matrix, which means that the volume fraction of any non-matrix phases is below the detectability of the X-ray method. The peaks were indexed according to their (hkl) reflection (minor peak denoted by a star belongs to the (111) K_β_ reflection). The broadening of the peaks obtained on the final CSET processed tube can be explained by the reduction in its crystallite size and by a microstrain introduced into the material. Rietveld refinement analysis showed a mean volume crystallite size Lvol-IB of about 85 nm and a microstrain of $\varepsilon_{0}$ ≈ 0.036.

### 3.2. Characterisation of Mechanical Properties

**Figure 9:**
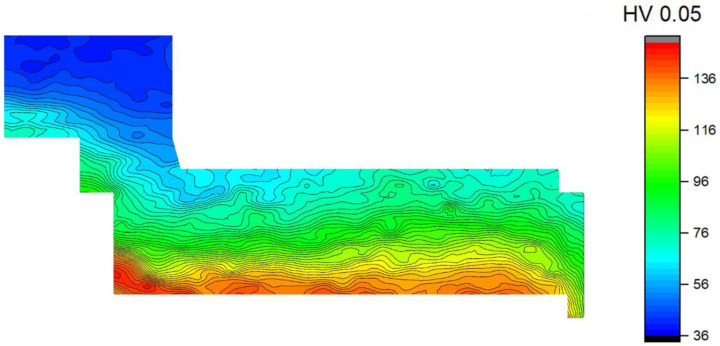
The variation of the microhardness values through the MDZ. The length of the top (blue) edge is 30 mm.

Mechanical properties of the CSET-processed sample were tested through microhardness measurements. The alteration in the microstructure along the cross-section of the MDZ was also reflected in the variation of the microhardness through the MDZ. The Vickers microhardness map was measured on the ND plane in the MDZ, using a square grid with an indentation distance of 200 µm. The resulting map is shown in Figure 9.

The microhardness in the area in front of the extrusion, affected only by the press, exhibited an average value of 45 HV0.05, which is only slightly larger than the value measured at the annealed sample (40 ± 2 HV0.05). The microhardness values were found to increase with the passage of the material in the CSET canal, and higher values were found everywhere in the vicinity of the mandrel. The maximum values measured close to the rotating mandrel were close to 150 HV0.05. The microhardness in the tube wall was stated to be 120 HV0.05 and no gradient from the inner to the outer edge was detected.

## 4. Discussion

### 4.1. Estimate of the Strain Induced during Individual Stages of CSET Process

**Figure 10:**
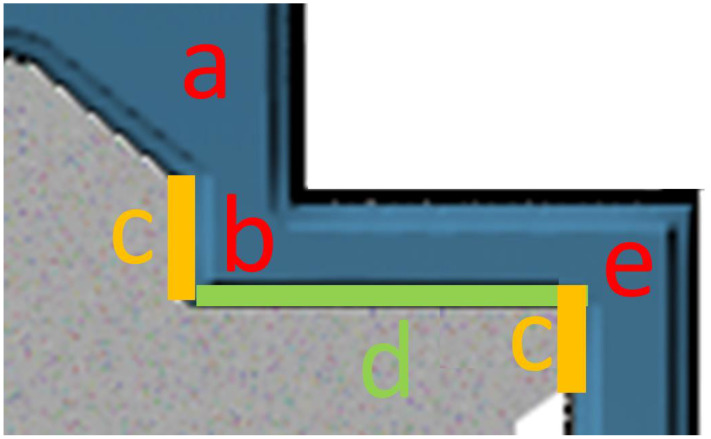
Detail of the MDZ on Figure 1b presenting the location where the individual stages of severe plastic deformation occur. The meaning of areas (**a**–**e**) is given in the text above the Figure.

CSET represents a rather complicated processing route enabling formation of the fine-grained tube directly from the initial cylindrical billet. It involves several stages of severe plastic deformation, which are indicated in schematic Figure 10:

(a) Extrusion (a);
(b) 1st ECAP pass 90° combined with circumferential strains (b,c);
(c) Shear deformation of the vertical part of the tube resembling HPTT (c);
(d) Shear deformation of the horizontal part resembling HPT (d);
(e) 2nd ECAP pass 90° (e).

The stages b and d of shear deformation are introduced by the rotation of the mandrel. The following part brings a rough estimate of contributions of individual stages to the cumulative strain.

The first deformation process during CSET is the extrusion of the billet around the mandrel tip. The equivalent strain for extrusion processes can be expressed as [16,17]

(1) $$\varepsilon_{extrusion}=\frac{2}{\sqrt{3}}ln\left(\frac{A_{1}}{A_{2}}\right)=\frac{2}{\sqrt{3}}ln\left(\frac{R_{billet}^{2}}{R_{outer}^{2}-R_{inner}^{2}}\right),$$

where *A*_1_ is the area of the initial cross-section of the sample, *A*_2_ denotes the cross-section after extrusion, *R_billet_* means the radius of the initial billet, and *R_outer_* and *R_inner_* are the outer and inner radii of the produced tube, respectively. By applying Equation (1) to the CSET form used in our experiment, the equivalent strain of 0.6 can be estimated using the parameters from Figure 1 (*R_billet_* = 5.5 mm, *R_outer_* = 5.5 mm, *R_inner_* = 3.5 mm).

After the extrusion step, the material goes through two ECAP passes. The equivalent strain for ECAP can be expressed as [1]

(2) $$\varepsilon_{ECAP}=\frac{N}{\sqrt{3}}\left[2\cot\left(\frac{\Phi +\Psi }{2}\right)+\Psi cosec\left(\frac{\Phi +\Psi }{2}\right)\right],$$

where *N* is the number of ECAP passes, Ψ and Φ are the outer and inner angle of ECAP channel, respectively; in our case, Φ = 90°and, for simplification, Ψ *=* 0°. The equivalent strain from one ECAP pass in this case then can be estimated as 1.15. However, during the passage of the material through the ECAP part, there were not only radial but also circumferential strains which lead to an additional strain increment, which modifying Equation (2):

(3) $$\varepsilon =\frac{2}{\sqrt{3}}ln\left(\frac{R_{outer1}^{2}-R_{inner1}^{2}}{R_{outer2}^{2}-R_{inner2}^{2}}\right),$$

and results in an equivalent strain of 1.13 (*R_outer_*_1_ = 5.5 mm, *R_inner_*_1_ = 3.5 mm, *R_outer_*_2_ = 13 mm, *R_inner_*_2_ = 11 mm).

Thus, the total equivalent strain from the translational movement of the material through the CSET is 4.03.

The simultaneous rotation of the mandrel leads to shear deformation due to the friction between the mandrel and the sample surface. The effect of the friction has to be evaluated with regard to the mutual directions of the sample mandrel surface orientation and translational movement of the material. The vertical part of the mandrel tip introduces a shear deformation (due to its rotation), just like it is during the HPTT process. Thus, the equivalent strain can be expressed in terms of HPTT as [18]

(4) $$\varepsilon =\frac{\theta }{\sqrt{3}ln\frac{R_{out}}{R_{in}}},$$

where *θ* is the angle of rotation in radians, and *R_out_* and *R_in_* are the outer and inner radii of the processed area, respectively.

In the case when no slip between the mandrel and the processed material occurs, a whole rotation (*θ* = 2π) leads to a strain value of 2.76 (*R_out_* = 13 mm, *R_in_* = 3.5 mm). Considering the translational and rotational velocity during CSET processing and assuming that no slip occurs, a maximum of 3 whole turns can be made.

A similar area can be found also at the end of MDZ, after the second ECAP pass, where a strain of 21.7 can be introduced into the material during a single complete turn for similar reasons (now *R_out_* is 13 mm, *R_in_* is 11 mm). However, due to the translational and rotational velocity during CSET processing, max 0.3 turns can occur.

The rotational movement of the mandrel tip leads also to a HPT-like process in the horizontal direction occurring between the two ECAP passes (stage d). The equivalent strain for the HPT is expressed as [1]:

(5) $$\varepsilon =\frac{\theta r}{\sqrt{3}h},$$

where *θ* is the angle of rotation in radians, *r* represents the distance from the centre of the disk, and *h* is the thickness of the deformed plate.

Supposing there is no slip between the material and the mandrel during processing, for a whole turn (*θ* = 2π), a total strain would be 8.7. Considering the translational and rotational velocity during CSET processing, up to 7.5 whole turns can appear. This estimate shows that the strain connected with mandrel rotation might be substantial. As discussed below, the press in the CSET form is nevertheless not high enough to avoid slip between the rotating mandrel and the flowing material. In reality, only a part of the mandrel rotation is transferred into the material. A more precise determination of this strain will be solved in our following research.

### 4.2. Microstructural Evolution of the 3003 Alloy during Given Stages of CSET Process

Different methods of severe plastic deformation are used for the enormous grain refinement of materials resulting in an increase in strength caused by a high density of grain boundaries. The microstructure evolution during severe plastic deformation has some common features. At the beginning, an increase in dislocation density is observed. During following stages of severe plastic deformation, the dislocations are arranged into low energy structures and, consequently, a fragmentation of initial microstructure followed by dynamic recovery occurs. The LAGBs are replaced by HAGBs, thus forming a submicrocrystalline structure from the initial millimetre-sized grains. A similar microstructure evolution was also expected during CSET.

The choice of experimental material was very important in our research. A sufficient RT plasticity on the one hand, and the stability of the ultra-fine-grained structure on the other hand are needed. The 3003 aluminium alloy with a low content of Mn and Cu was chosen for our experiment, especially on the basis of previous experiments with ECAP-processed samples of this alloy [19,20,21]. This material choice enabled us the CSET processing at RT and hindered grain coarsening of CSET processed tubes.

The main aim of the present research was to investigate the microstructural evolution during individual stages of the CSET process. At the start of the CSET process, the cast 3003 aluminium alloy billet was pressed by the plunger. This led to the generation of dislocation tangles already before extrusion (Figure 6a). In the following stage, the alloy passed through extrusion around the mandrel tip. Extrusion is usually carried out at elevated temperatures, which facilitates the process, but simultaneously an undesirable grain growth can occur [22,23]. Fortunately, the low-strength alloys exhibiting high ductility, such as 3003 aluminium alloy, are easy to extrude already at RT [24]. A relatively low temperature in the present case helped to reduce recovery processes and to retain a high strain in the sample. Extrusion led to spreading of the material around the mandrel tip accompanied with deformation of the grains of the processed material (Figure 3b). The crystallographic orientations of grains became more diffuse around a main orientation, proving an enhanced dislocation density in the area whose presence was confirmed by EBSD (Figure 3b) and TEM (Figure 6a). A layer of elongated refined grains was observed only in close vicinity of the mandrel tip (Figure 3c) which testifies for the enhanced stored deformation at this place due to the effect of rotating mandrel. The width of the grains was reduced below 1 μm, the aspect ratio was close to 5.

CSET includes two ECAP passes with channels intersecting at 90°. It was previously reported for the 3003 aluminium alloy that the first ECAP pass led only to increased dislocation density [25]. After the second ECAP pass of C route at RT, a high number of subgrains with an average size of 300–400 nm was formed [26]. Four ECAP passes were reported to lead to the formation of subgrains with a mean size of less than 500 nm [19,20,27]. Another Al–Mn alloy processed by four ECAP passes of C route at RT was reported to exhibit a grain refinement from 150 μm to 500 nm [24]. Further ECAP passes did not lead to further significant grain refinement; after eight ECAP passes at RT, the grain size of an Al–Mn-based alloy was only slightly smaller than 500 nm [28]. Thus, approximately, 4 to 8 ECAP passes (with accumulated equivalent strain between 4.6 and 9.2) were needed to reduce the grain size below 500 nm. If we compare these literature data with our results, we can see a similar microstructure evolution after the first ECAP pass at the position F, i.e., far from the rotating mandrel (Figure 2 and Figure 3f). However, a refinement by three orders of magnitude was observed at the position G in the middle of the sample (Figure 2 and Figure 3g, Table 2). Even faster grain refinement was observed at the position H, i.e., in vicinity of the rotating mandrel (Figure 2 and Figure 3h, Table 2). Table 2 also shows that the proportion of HAGBs increases significantly towards the side which is in contact with rotating mandrel. It is clear that the mandrel rotation brings a significant contribution to the equivalent strain.

The rotational movement of the mandrel leads to torsional straining of the processed material by two different processes—in the vertical parts of the processed sample, the deformation process is similar at the HPTT process, whereas the horizontal part of the sample in the MDZ area undergoes straining similar to that of HPT.

The vertical parts of the rotating mandrel realized HPTT-like deformation in the MDZ area. During classic HPTT, the tubular sample is placed between an internal and external mandrel, and an axial pressure is applied [11]. The rotation of the external mandrel leads to shear deformation in the wall, with a shear plane in a normal direction with respect to the tube radius, and a tangential shear direction. Similar to HPT, in the case of HPTT, the imposed strain also varies with the distance from the centre leading to a gradient microstructure. Aluminium of commercial purity subjected to 180° rotation of HPTT exhibited a final grain size of 650 nm, which was reduced from an initial size of 300 µm, after applying a shear of 20 [11]. An even more intensive grain size refinement was reported to occur during HPTT of an Al5086 alloy. A half-turn reduced the grain size to 100 nm after the applied shear of 20. The estimate of the contribution of this process to the equivalent strain during CSET described in Section 4.1 yields relatively high values of equivalent strain. However, this value is strongly overestimated. During HPTT, a large hydrostatic pressure is applied to the inner mandrel so that the tube sample is toughly pressed to the inner and outer mandrel, no slip occurs between the tube and both mandrels, and the tube is twisted. During CSET, the applied stress is much lower, the material flows through the CSET form, and a considerable slip occurs.

A similar overestimation of the strain can also be expected for the horizontal part of the sample. In HPT, a disc-shaped sample is subjected to torsional straining by placing between two anvils, where one or both are rotated. During processing, a high hydrostatic pressure is maintained to minimize slip between the rotating anvil and the sample. In HPT, the introduced strain varies with the distance from the disc centre. The microstructure in the central area usually resembles the material’s initial microstructure, whereas the edge region is the most influenced one. A single rotation already led to a grain size of around 1.5 μm in a high-purity pure Al sample processed under 1 GPa at RT [29]. Aluminium of commercial purity processed by HPT under 1 GPa exhibited a grain size decreasing from 0.5 mm to 1 µm [30]. In the case of CSET process, a significant slip can be expected because of much lower applied stress. Additionally, the sample undergoes an additional translational movement during CSET, which reduces the introduced strain but, simultaneously, ensures a more homogeneous strain distribution.

Homogenization of the microstructure occurs after the second ECAP pass, at the end of the CSET process. The grain size determined from EBSD maps is below 1 µm independently of the position through the tube wall (Figure 4). TEM experiments confirmed the presence of slightly elongated grains with the width of hundreds nanometres. The majority of grain boundaries have a high-angle character which corresponds well with materials prepared using other methods of SPD.

Individual stages of CSET also influence the grain shape. Markedly elongated grains were observed after extrusion in the direction of material flow (Figure 3c). These elongated grains contain low-angle boundaries which divide them into subgrains. The directionality of grains was observed also after the first ECAP pass in the close vicinity of the rotating mandrel (Figure 3d). The grains are inclined with their longer axis by 45° to the ECAP direction. It agrees well with the literature [19,27] showing the formation of the inclined shear bands which consist of elongated subgrains after the first ECAP pass. Nearly equiaxed grains were observed at the inner surface of the horizontal part of the processed sample beyond the first ECAP (Figure 3h). Such a microstructure change clearly documents the influence of rotating mandrel as completely different microstructure with not well-developed grains was observed at the outer surface (Figure 3f). Finally, the second ECAP pass leads again to a grain elongation; in this case, it is nearly parallel to the tube axis (Figure 3j and Figure 4). It seems that the grains have a pancake shape with the smallest size in the radial direction.

It was well documented in the literature that SPD leads to the significant strengthening of materials [1]. In the case of ECAP, the main strengthening occurs already during the first pass, while other passes were reported to have a minor contribution to overall strengthening (e.g., [25]). This reflects the fact that high density of dislocations is introduced into the material during the first ECAP pass, so that the dislocation strengthening especially determines the material strength. During the following ECAP passes, recovery processes result in the arrangement of dislocation into lower-energy structures followed by the formation of sub-boundaries, of LAGBs, and eventually of HAGBs. The strengthening effect of dislocations decreases; however, simultaneously, the strengthening effect of grain boundaries increases. To follow the strength changes during individual stages of the CSET process a very dense net of microhardness measurements was applied (Figure 10). The microhardness values successfully reflect the gradual transition of the coarse-grained microstructure of the annealed alloy toward the ultra-fine-grained microstructure in the tube wall. The microhardness measured in the top part of the MDZ (45 HV0.05, area A in Figure 2b) was slightly higher than that measured in the annealed sample (40 HV0.05), whereas the grain sizes were similar. This can be explained by increased dislocation density in area A caused by the pressing plunger prior to extrusion. Extrusion and two ECAP passes during CSET enhanced microhardness values by more than 100%. This increase is higher in comparison with pure ECAP, a hardness improvement by 68% was found after two passes of C type (following passes led to an even slower increase), and a 72% increase was observed after four passes of B type in an Al–Mn alloy [21]. The highest microhardness values around 140 HV0.05 were found at the inner surface of the horizontal part of the processed sample between both ECAP passes. This enhanced value can be explained by the influence of mandrel rotation (deformation process resembling HPT) and corresponds well to the ultra-fine-grained and equiaxed microstructure observed in this region. For comparison, the HPT of a commercial purity Al processed by eight turns of HPT under 1 GPa also led to an increment of almost three times [30]. It is clear from this comparison that the CSET process is very efficient, both in the formation of an ultra-fine-grained microstructure and in the improvement of the strength characteristics.

## 5. Conclusions

The conclusions of this paper can be summarized as follows:

- The capability of the CSET method to produce a tubular sample with ultra-fine-grained microstructure from an annealed cast alloy in one step was presented.
- The role of individual deformation steps, as extrusion, ECAP, and rotation-inducing torsional straining were presented and discussed.
- The individual deformation processes involved in CSET were demonstrated to result in gradual refinement of the microstructure.
- The main refinement occurred already after extrusion and the first ECAP step, where a grain refinement of three orders of magnitude occurred and the proportion of HAGB increased from only 17% to 72% in the middle of the wall.
- The highest refinement was achieved by the additional effect of the rotating mandrel. After the second ECAP step, the grain size was refined to approximately 500 nm and the proportion of HAGBs reached 90%. The microstructure is homogeneous through the tube wall.
- The microhardness of the final CSET processed tube was around 120 HV0.05, i.e., nearly three times higher in comparison with the initial material.

## Figures and Tables

**Table 1:** Positions in the processed sample where the microstructure were examined.

Position	Stage of the CSET Process	Position in the Tube
A	before extrusion, only pre-pressed	bulk
B	during extrusion	outer side, far from rotating mandrel
C	during extrusion	inner side, close to rotating mandrel
D	after extrusion	inner side, close to rotating mandrel
E	during the 1st ECAP pass	middle of the tube wall
F	after the 1st ECAP pass	outer side, far from rotating mandrel
G	after the 1st ECAP pass	middle of the tube wall
H	after the 1st ECAP pass	inner side, close to rotating mandrel
I	during the 2nd ECAP pass	outer side, far from rotating mandrel
J	after the 2nd ECAP pass	middle of the tube wall

**Table 2:** Microstructural parameters (grain width and length, grain aspect ratio, and percentage of HAGBs evaluated from the OIMs in Figure 3) taken from positions denoted in Figure 2.

Position	Grain Width [µm]	Grain Length [µm]	Grain Aspect Ratio	HAGB [%]
C	0.8	4	5	80
E				<20
F				35
G	1	2	2	70
H	0.4	0.4	1	90
I				60
J	0.4	0.8	2	90

## Data Availability

This article has no additional data. All data included in this study are available upon request by contact with the corresponding author.
